# Implementation of active therapeutic hypothermia across a regional transport network for infants transferred for neonatal encephalopathy

**DOI:** 10.1038/s41390-025-04248-x

**Published:** 2025-06-27

**Authors:** Aarti Mistry, Nora Imolya, Jack Fletcher, Dharmapuri Sobithadevi, Davina Sham, Benjamin James Baucells, Julia Edwards, Arthi Lakshmanan, Andrew Currie, Andrew Leslie, Shalini Ojha, Don Sharkey

**Affiliations:** 1 Centre for Perinatal Research (CePR), School of Medicine, University of Nottingham, Nottingham, UK; 2 Nottingham Centre for Neonatal Care, Nottingham University Hospitals NHS Trust, Nottingham, UK; 3 Neonatal service, University Hospitals Derby and Burton NHS Trust, Derbyshire, UK; 4 CenTre Neonatal Transport Service, University Hospitals of Leicester NHS Trust, Leicester, UK; 5 Neonatal Service, University Hospitals of Coventry and Warwickshire NHS Trust, Coventry, UK

## Abstract

**Background:**

The aim of this study was to evaluate the introduction of servo-controlled therapeutic hypothermia (TH) by a regional transport service and referring centres for infants with hypoxic ischaemic encephalopathy (HIE). The primary objective was to compare the time to reach 33–34 °C target temperature (TT).

**Methods:**

This is a retrospective cohort study of neonatal transfers for TH across a large UK regional network from 2011 to 2021. Three cohorts were identified for comparison, defined by the setting of TH initiation: referring ‘Base’ centre or during ‘Transport’ and by the TH method: passive^(Pass)^ or active^(Act)^.

**Results:**

A total of 315 infants were included. The Transport^Act^ (*n* = 128) cohort achieved TT significantly earlier (280 min) than the Transport^Pass^ (*n* = 155) cohort (353 min, *P* < 0.001), with 84% vs 46% (OR 6.3, 95% Cl 3.3–11.8, *P* < 0.0001) achieving this within 6 h of birth. Introduction of Base^Act^ (*n* = 32) was associated with an additional 89 min reduction in time to TT (191 min, *P* < 0.0001), with more infants achieving this within 3 h (44% vs 19%; OR 3.3, 95% Cl 1.4–7.7, *P* < 0.01), and a shorter stabilisation time (110 vs 175 min, *P* < 0.001). Outcomes for infants were not different.

**Conclusions:**

Compared with passive cooling, the introduction of transport and referring centre active TH improves temperature management of transferred infants with HIE, with more reaching therapeutic temperature within 6 h and fewer being overcooled.

**Impact:**

- Early therapeutic hypothermia (TH) improves survival without disability for infants with hypoxic ischaemic encephalopathy (HIE). However, many infants are born in centres without servo-controlled TH and rely on passive cooling prior to transfer.

- This study demonstrates that the introduction of active TH by both the transport team and by referring centres is associated with significant improvements in time to reach target temperature, minimising overcooling and reducing transport stabilisation times.
- Investment in active TH provision by all birth centres and transport teams could be a cost-effective method to reduce birth-related brain injury and improve outcomes of infants with HIE.

## Introduction

Hypoxic ischaemic encephalopathy (HIE) is a major cause of term brain injury globally with an incidence of 1–2/1000 live births.^1^ In the UK, approximately 500 infants per year with HIE require inter-hospital transfer for centralised intensive care with therapeutic hypothermia (TH).^2^ For infants with moderate/severe HIE, TH between 33 and 34 °C improves survival without disability when commenced within 6 h of birth, known as ‘the therapeutic window’.^3,4^

Active TH provided by a servo-controlled device is the optimal method for delivering TH^5^ compared to passive cooling,^6^ where there is a greater risk of overcooling (<33 °C) and potential harm.^7,8^ Servo-controlled TH is usually provided by neonatal intensive care units (NICUs), i.e. tertiary cooling centres, often resulting in passive cooling initially for infants with HIE born in non-cooling centres.

The safety and effectiveness of servo-controlled TH during neonatal transport^9–12^ has resulted in UK transport services adopting its use ahead of other countries.^2,13,14^ Infants with HIE born in non-cooling centres are at greater risk of poorer neurological outcomes compared to inborn infants.^15,16^ Globally, there is significant variability in the pathway of care for these infants, making it challenging for them to receive optimal treatment within 6 h.^13,17,18^ These disparities are a consequence of centralisation of neonatal care, regional variations in access to equipment, healthcare infrastructure, and geographical barriers.^13,18,19^

In the UK, the national HIE framework recommends that all levels of maternity/neonatal units should have access to servo-controlled TH equipment at birth, to enable earlier TH initiation.^20^ However, marked regional variation in the provision of active TH at birth exists.^21^ It is unclear if differences in the provision of active TH affect HIE therapeutic targets, transport metrics and short-term NICU outcomes for infants transferred for HIE. Understanding the impact of transport on infants requiring transfer for HIE and their thermoregulatory management were both identified as top ten neonatal transport research priorities.^22^

Our study aimed to understand how the implementation of servo-controlled TH by a regional transport service and adoption by referring centres affect important TH metrics. The primary objective was to compare the time to reach the 33–34 °C target temperature (TT). Secondary objectives explored the impact on transport process metrics and short-term infant outcomes.

## Methods

### Population

The Trent Perinatal and Central Neonatal Network (TPCNN) consists of 14 neonatal units; four NICUs (level 3) providing active TH, five Local Neonatal Units (level 2) and five Special Care Baby Units (level 1) (Supplementary File, Fig. 1). The CenTre Neonatal Transport Service provide transfer services across this network, undertaking 1298–1660 transfers per year, of which 2–3.3% are infants with HIE (Supplementary File, Table 1).

CenTre Neonatal Transport Service commenced use of servo-controlled TH devices in 2015; prior to this, infants were passively cooled in line with the procedure used in the TOBY trial.^23^ In 2016 and 2019, two Level 2 units switched from passive cooling to servo-controlled TH devices. The regional transport service and referring centres used the CritiCool servo-controlled TH device with the infant CureWrap (Belmont Medical Technologies, Billerica, Massachusetts). Changes in utilisation of dedicated crews and ambulances over the study period are outlined in Supplementary File, Table 2. Transfers of infants with HIE were considered as time-critical requiring team mobilisation within 60 min from base following referral.^14,24^

Infants referred for cooling transfer from April 2011 to March 2021 were identified from the routinely recorded transport database, and duplicated records were removed. All infants referred within TPCNN to the regional transport service for cooling transfer and completed transfer to a TPCNN cooling centre for ongoing management for HIE were included. The clinical decision to commence cooling was made by the referring centre and reviewed by the transport team on arrival. The referring centres and transport team followed a regional guideline to ensure that the infant met diagnostic criteria defined in the TOBY trial^23^ for initiating TH and for continuing TH treatment for HIE, warranting transfer to a regional cooling centre for ongoing care. The regional TH protocol includes that all infants ≥36 weeks gestation should be discussed for cooling transfer, and infants between 34 and 35 + 6 weeks gestation may be considered for cooling transfer at the discretion of the accepting tertiary cooling centre team.^25^

Infants not completing transfer, as cooling was no longer indicated after transport team assessment or the infant died before transfer, were excluded, along with infants referred from or completing transfer to a cooling centre outside of the TPCNN. Infants were split into one of three cohorts based on the combination of cooling method at their Base hospital (referring centre) and during Transport, with either passive^(Pass)^ or active^(Act)^ TH:

Cohort (1) Base^Pass^/Transport^Pass^ (passive TH by both referring centre and transport);

Cohort (2) Base^Pass^/Transport^Act^ (passive TH by referring centre/active TH by transport);

Cohort (3) Base^Act^/Transport^Act^ (active TH by both referring centre and transport team).

### Data collection

The change in access to active TH in the TPCNN resulted in this evaluation to understand the impact on the care pathway for infants with HIE. The service evaluation was registered with the University Hospitals of Leicester clinical audit department. The transport and tertiary team leads agreed to undertake infant data collection and co-developed the data collection proforma. Anonymised data were collected from routinely recorded clinical data in the transport database and local electronic record as detailed in Supplementary File, Table 3.

Infant characteristics included demographics, antenatal, intrapartum and resuscitation details. The primary objective was to establish the time to reach TT and achievement within 6h of age and TT at each stage of the transport pathway. Secondary objectives centred around transport team metrics including mobilisation and stabilisation times. Infant management and short-term outcomes on the NICU were also collected, definitions are outlined in the Supplementary File, Table 3. Standard magnetic resonance imaging (MRI) protocols were used consisting of T1, T2 and diffusion-weighted imaging (DWI) and reported locally by paediatric radiologists.

### Statistical analysis

The final database was analysed using Stata SE V18 (StataCorp, College Station, Texas). Data returned as ‘unknown’ or incomplete were classified as ‘missing data’. Statistical significance was calculated using Chi-squared test and odds ratios (ORs) with 95% confidence intervals (Cls). For non-parametric, continuous data, a two-tailed Mann–Whitney test was used to compare medians. Statistical significance was defined as *P* < 0.05 and all data were unadjusted. Comparisons were made between cohorts 1 and 2 and between cohorts 2 and 3. Missing data were reported as a percentage of the total denominator for all cohorts combined.

## Results

**Fig. 1:**
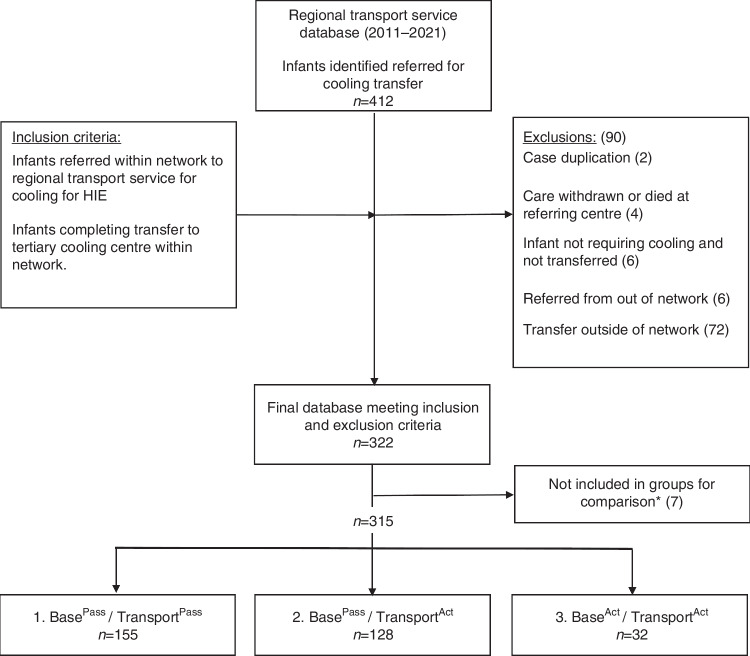
Consort table of inclusion and exclusion criteria. Regional network refers to the infant referred within the Trent Perinatal and Central Neonatal Network. Three groups for comparison defined: (1) Base^Pass^/Transport^Pass^ (passive therapeutic hypothermia (TH) by both the referring centre and Transport team), (2) Base^Pass^/Transport^Act^ (passive TH by referring centre and Active TH by transport team), (3) Base^Act^/Transport^Act^ (active TH by both referring centre and Transport team). *Infants did not meet the definitions of the final predefined groups for comparison.

A total of 394 infants were referred from within the TPCNN of which 322 were included in the analysis (Fig. 1). Exclusions included 10 infants who did not complete transfer and 72 who were transferred outside of the TPCNN network.

Of the 322 infants, the median age of referral to the transport service was 131 min (interquartile range (IQR) 88–190). Irrespective of the method of TH, 212 (67.7%) achieved TT within 6 h, and 245 (77.9%) arrived at the cooling centre within TT.

On admission to the cooling centre, 263 (81.9%) completed 72 h of TH. Of the 58 infants who did not complete full TH treatment, 22 died, 30 were considered to have mild HIE and 6 required significant cardiorespiratory support, leading to discontinuation of TH.

For the cohort analyses, seven infants were excluded as they did not fit into the defined groups, leaving 315 infants for comparison (Fig. 1 and Table 1). A total of 283 infants received Base^Pass^ prior to transport, of which 155 received Transport^Pass^ and 128 received Transport^Act^; 32 infants received both Base^Act^ and Transport^Act^. Six infants, two in each comparison group, were 34 + 0–35 + 6 weeks gestational age. The median transfer distances across comparison groups ranged between 34.4 and 52.5 km, with no difference observed between groups (Supplementary File, Table 4).

**Table 1:** Infant characteristics of three cohorts: (1) Base^Pass^/Transport^Pass^, (2) Base^Pass^/Transport^Act^ and (3) Base^Act^/Transport^Act^.

Infant characteristics	(1) Base^Pass^/Transport^Pass^ *n* = 155; *n* (%)	(2) Base^Pass^/Transport^Act^ *n* = 128; *n* (%)	(3) Base^Act^/Transport^Act^ *n* = 32; *n* (%)	Missing *n* (%)
Median (IQR) gestation (weeks)	40 (38, 41)	39 (38, 40)	40 (37.5, 40)	–
Median (IQR) birth weight (g)	3395 (2835, 3770)	3280 (2785, 3680)	3360 (3018, 3523)	–
Sex (female)	76 (49.0)	56 (43.8)	15 (46.9)	–
Singleton	146 (94.2)	121 (94.5)	31 (96.9)	–
Referring centre				
SCBU (Level 1)	81 (52.3)	60 (46.8)	0	–
LNU (Level 2)	67 (43.2)	66 (51.6)	27 (84.4)	
NICU (Level 3)	7 (4.5)	2 (1.6)	5 (15.6)	
Antenatal risk factors				
IUGR	27 (17.4)	22 (17.2)	5 (15.6)	–
Large for gestational age	19 (12.3)	18 (14.1)	3 (9.4)	–
Reduce fetal movements	15 (10.6)	11 (8.7)	4 (12.5)	14 (4.4)
Preeclampsia/PET	10 (6.9)	5 (3.9)	1 (3.1)	11 (3.5)
Gestational/IDDM	2 (1.4)	13 (10.2)	5 (15.6)	16 (5.1)
Antepartum haemorrhage^a^	20 (13.3)	17 (13.3)	4 (12.5)	5 (1.6)
Maternal infection^b^	25 (18.4)	12 (9.8)	6 (18.8)	24 (7.6)
PROM	30 (20.6)	14 (11.3)	6 (18.8)	13 (4.1)
Mode of delivery				
Normal vaginal delivery	41 (26.6)	42 (32.8)	6 (18.8)	1 (0.32)
Instrumental	34 (22.1)	19 (14.8)	6 (18.8)	
EL-LSCS not in labour	3 (1.9)	5 (3.9)	16 (50)	
EM-LSCS not in labour	23 (14.9)	21 (16.4)	2 (6.3)	
EM-LSCS in labour	53 (34.4)	41 (32.0)	2 (6.3)	
Presentation at birth				
Cephalic	133 (88.7)	106 (86.9)	25 (80.7)	12 (3.8)
Breech/other	17 (11.3)	16 (13.1)	6 (19.3)	
Onset of labour				
None	22 (14.3)	23 (18.0)	4 (12.5)	1 (0.32)
Spontaneous	103 (66.9)	73 (57.0)	19 (59.4)	
Induced	29 (18.8)	32 (25.0)	9 (28.1)	
Intrapartum events				
Shoulder dystocia	9 (6)	12 (9.8)	3 (9.4)	11 (3.5)
Cord prolapse	4 (2.7)	7 (5.7)	1 (3.1)	12 (3.8)
Malposition	16 (10.6)	10 (8.1)	5 (15.6)	9 (2.9)
Failure to progress	20 (13.7)	13 (10.7)	3 (9.7)	17 (5.4)
Fetal distress in labour	73 (53.3)	59 (52.7)	14 (45.2)	35 (11.1)
Fetal bradycardia	60 (44.4)	57 (51.8)	17 (54.8)	39 (12.4)
Significant meconium	55 (36.2)	42 (33.6)	8 (25)	6 (1.9)
Resuscitation				
None	6 (3.9)	2 (1.6)	1 (3.1)	–
Mask ventilation	45 (29)	44 (34.4)	14 (43.8)	–
Intubation	104 (67.1)	82 (64.1)	17 (53.1)	–
Chest compression	56 (36.4)	44 (34.4)	8 (25)	1 (0.32)
Drugs— adrenaline	17 (11)	12 (9.4)	4 (12.5)	1 (0.32)
Apgar @ 1 min, Median (IQR)	2 (1, 3)	2 (1, 4)	2 (0, 2)	9 (2.9)
Apgar @ 5 min	5 (3, 6)	4 (3, 6)	4 (3, 5)	9 (2.9)
Apgar @ 10 min	6 (4, 8)	6 (4, 7)	6 (4, 7)	37 (11.7)
Cord venous pH, Median (IQR)	7.10 (6.94, 7.22)	7.15 (7.02, 7.28)	7.11 (6.90, 7.28)	51 (16.9)
NICU management				
No respiratory support^c^	25 (16.1)	20 (15.6)	8 (25)	–
Non-invasive ventilation^d^	5 (3.2)	0	2 (6.3)	–
Mechanical ventilation	125 (80.7)	108 (84.4)	22 (68.5)	–
Days of ventilation, Median (IQR)	3 (2, 5)	3 (2, 5)	4 (3, 5)	–
Inotropes	57 (36.8)	45 (35.2)	7 (21.9)	–
Anticonvulsants	54 (35.1)	44 (34.4)	9 (28.1)	1 (0.32)
Reported grade of HIE				
Mild	53 (34.4)	35 (27.1)	14 (43.8)	1 (0.32)
Moderate	65 (42.2)	59 (46.1)	10 (31.3)	
Severe	36 (23.4)	33 (25.8)	8 (25)	
Other diagnosis	0	1 (0.78)	0	

Missing *n* (%) denominator of *n* = 315. Definitions of infant variables can be found within the Supplemental File, Table 3.

*SCBU* Special Care Baby Unit (Level 1), *LNU* Local Neonatal Unit (Level 2), *NICU* Neonatal Intensive Care Unit (Level 3), *IUGR* intrauterine growth restriction, *PROM* prolonged rupture of membrane greater than 18 h, *PET* preeclampsia toxaemia, *EM-LSCS* emergency caesarean section, *EL-LSCS* elective caesarean section, *HIE* hypoxic ischaemic encephalopathy.

^a^Antepartum haemorrhage includes placental issues of praevia or abruption.

^b^Maternal infection—chorioamnionitis, UTI, maternal presumed sepsis, maternal pyrexia >38.5 °C.

^c^No respiratory support includes nasal cannula oxygen.

^d^CPAP and high flow.

### Temperature outcomes

**Fig. 2:**
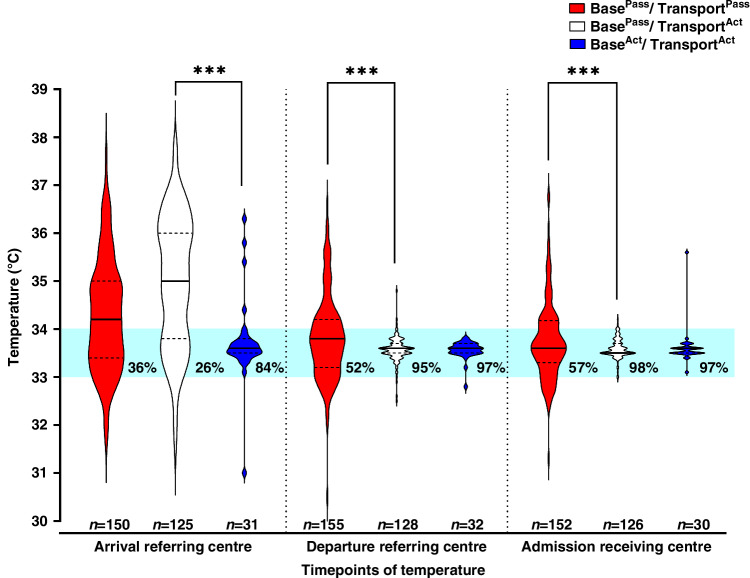
Violin plot of infants transferred for cooling and their temperature at transport team arrival and departure from the referring centre and admission to the receiving cooling centre. Percentage of infants in the therapeutic target temperature (TT) range (33–34 °C), which is depicted by the shaded area in blue. ***P < 0.001. Temperature data at each time point were not available for a small number of infants and are reflected in the relevant group numbers.

Temperature on arrival of the transport team at referring Base^Pass^ centres for Cohorts 1 and 2 were similar with overall 31.3% (86/275) of babies in the TT range (Fig. 2). Compared to the Base^Pass^ infants, significantly more Base^Act^ infants (83.9%, 26/31) were in the TT range when the transport team arrived (OR 11.4, 95% Cl 4.0–32.7, *P* < 0.001).

Implementation of Transport^Act^ resulted in significantly more infants departing the referring centre in the TT range with 95.3% (122/128) of infants vs 51.6% (80/155) with Transport^Pass^ (OR 19.1, 95% Cl 7.0–51.9, *P* < 0.0001). Fewer Transport^Act^ infants departed overcooled at <33 °C (2.3%, 3/128) compared with Transport^Pass^ (14.2%, 22/155) (OR 0.15, 95% Cl 0.1-0.5, *P* < 0.001). On arrival at the receiving centre, 98.4% (124/126) of Transport^Act^ infants were in TT on admission to the receiving cooling centre compared with only 57.2% (87/152) of Transport^Pass^ infants (OR 46.3, 95% Cl 9.0–237.9, *P* < 0.0001).

**Fig. 3:**
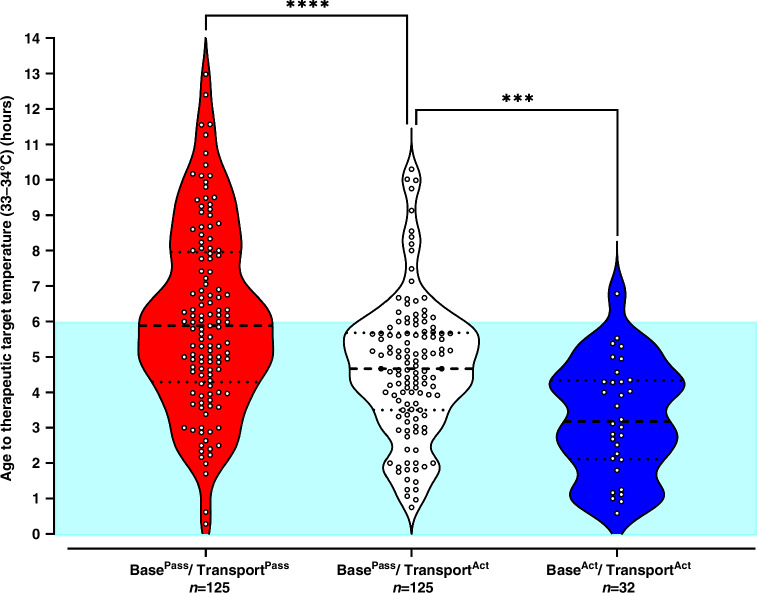
Violin plot of age to therapeutic target temperature (TT) of 33–34 °C is reached for cohorts (1) Base^Pass^/Transport^Pass^, (2) Base^Pass^/Transport^Act^ and (3) Base^Act^/Transport^Act^. Blue shaded area depicts the 6-h ‘therapeutic window’ from birth. Mann–Whitney test is used to compare cohorts. ***P < 0.001, ****P < 0.0001. Missing values:32/315(10.2%). One data point graphically not presented in cohort (1) Base^Pass^/Transport^Pass^, Age to TT value of 1339 min.

With the implementation of transport servo-controlled TH, the median age to TT significantly improved from 353 min (IQR 258–478) for Base^Pass^/Transport^Pass^ to 280 min (IQR 210–341) for Base^Pass^/Transport^Act^ infants (*P* < 0.0001, Fig. 3), resulting in 84.4% (108/128) vs 46.3% (68/147) achieving TT within 6 h of age (Table 2). The introduction of Base^Act^ further reduced the age to TT by 89 min with a median age of 191 min (IQR 127–260, *P* < 0.001). Of the 32 infants who received Base^Act^/Transport^Act^, 31 (96.9%) achieved TT within 6 h and were significantly more likely to achieve TT within 3 h of birth compared to infants receiving Base^Pass^/Transport^Act^ (Table 2).

**Table 2:** Key transport metrics for cohorts.

Transport outcomes/metrics	(1) Base^Pass^/Transport^Pass^ *n* = 155	(2) Base^Pass^/Transport^Act^ *n* = 128	(3) Base^Act^/Transport^Act^ *n* = 32	1 vs 2		2 vs 3		Missing *n* (%)
				OR (95% Cl)	*P* value	OR (95% Cl)	*P* value	
Infant age (min), median (IQR)								
At referral	129 (73, 195)	125 (88, 166)	189 (138, 258)	–	0.86	–	<0.0001	3 (1.0)
At the departing transport base	223 (153, 290)	180 (139, 231)	243 (222, 369)	–	<0.01	–	<0.0001	–
Arriving at the baby	281 (215, 362)	250 (200, 306)	320 (283, 443)	–	<0.01	–	<0.0001	–
Departure with a baby	447 (370, 545)	435 (370, 540)	505 (406, 590)	–	0.89	–	0.04	–
On admission to the tertiary cooling centre	510 (418, 611)	498 (419, 616)	563 (453, 647)	–	0.91	–	0.11	–
Transfer metrics (min), median (IQR)								
Mobilisation	70 (43, 115)	45 (32, 80)	59 (40, 134)	–	<0.001	–	0.04	3 (1.0)
Response	135 (95, 185)	115 (95, 151)	125 (99, 183)	–	0.04	–	0.12	3 (1.0)
Stabilisation	145 (105, 200)	175 (125, 238)	110 (93, 203)	–	<0.01	–	<0.01	–
Target temperature reached, *n* (%)								
<3 h of age	17 (13.5)	24 (19.2)	14 (43.8)	1.52 (0.77, 3.01)	0.22	3.27 (1.39, 7.69)	<0.01	32 (10.2)
<6 h of age	68 (46.3)	108 (84.4)	31 (96.9)	6.27 (3.34, 11.77)	<0.0001	5.74 (0.72, 45.41)	0.06	8 (2.5)
Transfer management, *n* (%)								
No respiratory support	40 (25.8)	27 (21.1)	15 (46.9)	0.77 (0.44, 1.34)	0.35	3.30 (1.43, 7.64)	<0.01	–
Non-invasive ventilation	5 (3.2)	0	1 (3.1)	–	0.04	–	0.04	–
Mechanical ventilation	113 (72.9)	101 (78.9)	16 (50)	1.39 (0.80, 2.42)	0.24	0.27 (0.11, 0.62)	0.001	–
Inotropes	38 (24.5)	22 (16.4)	3 (9.4)	0.60 (0.33, 1.09)	0.09	0.53 (0.15, 1.91)	0.32	–
Nitric oxide	6 (3.9)	6 (4.7)	0	1.22 (0.38, 3.89)	0.73	–	0.21	–
Seizure/anticonvulsants^a^	68 (44.7)	46 (35.4)	11 (34.4)	0.68 (0.42, 1.10)	0.12	0.95 (0.42, 2.16)	0.91	–

Comparisons between cohorts 1 and 2 and between cohorts 2 and 3 are presented. For categorical and binary outcomes, chi-square and odds ratios (ORs) and 95% confidence intervals (Cls) are presented. For non-parametric continuous data, Mann–Whitney test is used for comparison. Statistical significance is defined as *P* < 0.05. Missing *n* (%) based on denominator *n* = 315. Definitions: mobilisation time is the period from referral to departure from the transport base. Response time is the period from referral to arrival at the referring centre. Stabilisation time is the period from arrival and departure from the referring centre.

^a^Includes seizures clinical or electrical or use of anticonvulsants prior to transfer.

### Transport process metrics and infant outcomes

Following the introduction of active cooling for transport, teams were quicker mobilising and departing with a 43 min reduction in age to departing base (Table 2). Transport^Act^ transfers had a median increase in stabilisation time of 30 min. Compared with Transport^Pass^, Transport^Act^ resulted in absolute 38.1% increase in the number of infants achieving TT within 6 h of age.

The introduction of Base^Act^ was associated with a significant increase in the median age of referral, with transport teams arriving at the referring centre at a later age, with no significant changes in median mobilisation and response times. Significantly more infants receiving Base^Act^ reached TT within 3 h of age and had a reduction in stabilisation time of 65 min compared with Base^Pass^/Transport^Act^ cohort, although fewer required respiratory support. There were no differences in important short-term NICU infant outcomes across the three cohorts (Table 3).

**Table 3:** Infant outcomes across three cohorts.

Infant outcomes	(1) Base^Pass^/Transport^Pass^ *n* = 155	(2) Base^Pass^/Transport^Act^ *n* = 128	(3) Base^Act^/Transport^Act^ *n* = 32	1 vs 2		2 vs 3		Missing *n* (%)
				OR (95% Cl)	*P* value	OR (95% Cl)	*P* value	
NICU management, *n* (%)								
No respiratory support	25 (16.1)	20 (15.5)	8 (25)	0.96 (0.51, 1.83)	0.90	1.80 (0.70, 4.60)	0.21	–
Mechanical ventilation	125 (80.7)	108 (84.4)	22 (68.8)	1.29 (0.69, 2.42)	0.41	0.41 (0.17, 1.00)	0.04	–
Days of ventilation^a^, Median (IQR)	3 (2, 5)	3 (2, 5)	4 (3, 5)	–	0.41	–	0.68	–
Inotropes	57 (36.8)	45 (35.2)	7 (21.9)	0.93 (0.57, 1.52)	0.78	0.52 (0.21, 1.30)	0.15	–
Completed cooling 72 h	121 (78.6)	108 (84.4)	27 (84.4)	1.49 (0.8, 2.75)	0.20	0.99 (0.34, 2.88)	0.99	1 (0.3)
Intended to complete cooling	136 (88.3)	121 (94.5)	28 (87.5)	2.29 (0.92, 5.7)	0.07	0.4 (0.11, 1.49)	0.16	1 (0.3)
Seizures	58 (37.4)	54 (42.5)	9 (28.1)	1.22 (0.76, 1.97)	0.41	0.54 (0.23, 1.26)	0.15	–
Anti-convulsant	54 (35.1)	44 (34.4)	9 (28.1)	0.97 (0.59, 1.58)	0.90	0.75 (0.32, 1.76)	0.50	1 (0.3)
Brain injury on MRI	67 (49.3)	49 (41.2)	17 (58.6)	0.72 (0.44, 1.19)	0.19	2.02 (0.88, 4.67)	0.09	6 (2.1)^c^
Day MRI performed, Median (IQR)	6 (4, 9)	6 (5, 9)	5 (4, 5)	–	0.99	–	<0.001	21 (8.0)^c^
Discharge destination, *n* (%)								
Died	17 (11)	12 (9.4)	3 (9.4)	–	0.89	–	0.86	–
Home	135 (87.1)	113 (88.3)	29 (90.6)					
Hospice/other	3 (1.9)	3 (1.6)	0					
Length of stay^b^, Median (IQR)	9 (7, 12)	10 (8, 13)	9 (7, 14)	–	0.22	–	0.41	3 (1.1)^d^
Oral feeds at discharge^b^	127 (94.1)	102 (87.9)	26 (89.7)	0.45 (0.18, 1.14)	0.08	1.19 (0.32, 4.47)	0.79	3 (1.0)^d^
Survival outcomes, *n* (%)								
Survival at discharge	138 (89)	116 (90.6)	29 (90.6)	1.19 (0.55, 2.59)	0.66	1.00 (0.26, 3.79)	1.00	–
Survival with no brain injury	69 (50.7)	68 (57.1)	12 (41.4)	1.3 (0.79, 2.13)	0.30	0.53 (0.23, 1.22)	0.13	6 (2.1)^c^
Survival with no seizures	95 (61.3)	70 (54.3)	22 (68.8)	0.76 (0.47, 1.23)	0.26	1.82 (0.79, 4.19)	0.15	–

Comparison made between cohorts 1 and 2 and between cohorts 2 and 3. For categorical and binary outcomes, chi-squared and odds ratios (ORs) and 95% confidence intervals (Cls) are presented. For non-parametric continuous data, Mann–Whitney test was used. Statistical significance is defined as *P* < 0.05. Missing *n* (%) based on denominator *n* = 315.

^a^Infants who only received mechanical ventilation.

^b^Infants who survived at discharge.

^c^Number of MRI scans performed.

^d^Number of infants survived at discharge.

## Discussion

Across a large regional network, the implementation of servo-controlled active TH by transport teams significantly improves the hypothermic management of infants with HIE, in particular the attainment of TT within 6 h of birth. When this is combined with active TH at the referring centre, there is an even greater improvement in hypothermic management. Importantly, at the point of transfer, there was almost a sevenfold reduction in the number of infants who were overcooled compared to those for whom only passive cooling was available. Overall, active TH at the referring centre and during transport was associated with earlier TH, leading to more infants reaching TT sooner and more than 95% starting the transport journey in the desired target range.

Delivering optimal treatment to infants with HIE born in non-cooling centres and requiring transfer is a global challenge, and understanding the impact of transport on these infants is a research priority.^22^ Servo-controlled TH devices are now used by all UK transport teams^26^ but use is more variable in other developed countries.^13,14,18^ Our study shows active TH during transport is associated with a significant reduction in the age to TT and a greater proportion achieving this within 6 h, bridging the gap for infants previously exposed to prolonged periods of suboptimal passive cooling^6,12^ who were required to await admission to a cooling centre for active TH.^21^ Despite this, we still observe a proportion of infants not reaching TT within 6 h of birth. Transport factors that may contribute to the delay achieving TT include age of referral and availability of a dedicated ambulance and team to enable faster mobilisation and timely arrival to the referring centre.^27^ These elements are important, along with the method of TH being delivered by transport teams. Our data suggest the use of active TH at birth centres may help mitigate some of these factors by reducing the time pressure on transport teams to arrive within the therapeutic window of 6 h, particularly in situations when transport teams may not be available to despatch immediately. Whilst we observed shorter stabilisation times in referral centres with active TH, we cannot say for certain that this relates to this change in practice or if other factors are also involved, as this cohort of infants required less invasive ventilation, which may have reduced turnaround times.

Delivery of active TH in referring hospitals may offer better neuroprotection by enabling earlier initiation of TH, with more infants reaching TT within 3 h of age. Earlier TH within 3 h of birth has been associated with greater neuroprotection, compared with later initiation (3–6 h), with animal studies reporting less neuronal loss,^28–30^ and observational human studies suggesting improved motor development and fewer seizures.^31,32^ Seizures in the context of HIE are associated with poorer outcomes.^33,34^ We did not observe significant differences in the number of infants with seizures or survival without seizures in infants receiving Base^Act^, probably due to the small number of infants in this group.

The UK national HIE framework recommends all maternity/neonatal centres to have immediate access to servo-controlled TH.^20^ A recent study found 39% of UK births lacked this access at their birth centre and so relied on transport teams or cooling centres to initiate TH, with marked variation across regions.^21^ Gradual improvement in access has been observed, driven through quality improvement initiatives such as ‘Time=Brain’^35,36^; however, to standardise care for all infants requires further investment.

We show that access to TH equipment in both settings is an essential part of delivering efficient and optimal thermoregulatory care for infants transferred for HIE. This should be in parallel with education and training to support early identification and referral to transport teams to prompt discussion and guide decision-making. This approach is feasible in the UK, due to the neonatal healthcare and transport infrastructure^2,37^ and with the majority of transfers undertaken by road ambulance with a journey time under 2 h.^17^ For services covering larger geographical regions, with different transport modalities, or in settings with different healthcare infrastructure, limited neonatal expertise and resources at referring centres, this may be a less viable process.^13,14,18^

### Strengths

This is the first large regional study of how stepwise implementation of active TH by transport and referring centres impacts achievement of HIE TH targets, thermoregulatory management and important transport metrics. Previous studies have either focused just on TH during transport or were small cohorts or single centred^.^^10,11,38,39^ This regional analysis of HIE management provides supportive evidence towards the recommendations outlined in the national HIE framework published after this study period.^20^

### Limitations

The retrospective nature of the study limits our ability to control for data entry errors, incomplete records, missing data and potential reporting bias. To minimise these, we used standardised proformas and definitions and validated the returned data to check for inaccuracies.

Due to the small numbers within the comparison groups, our analysis did not adjust for potential confounders, and we did not observe any significant differences in short-term infant outcomes. A larger national study would be necessary to confirm any associations between improved thermal management and its impact on long-term neurodevelopmental outcomes and cost-effectiveness. Furthermore, a formal health economic evaluation and estimation of resource utilisation would be important to consider in future studies but is beyond the scope of this report.

The variability in MRI timing and reporting by different clinicians across centres could lead to inconsistencies in the reporting of scan findings.^40,41^ MRIs performed beyond day 7 of life can reduce the sensitivity of identifying hypoxic brain injury, especially on DWI and T2 scans.^41,42^ Paediatric radiologists reported the scans, but as a pragmatic service evaluation, we did not standardise the reports to any published scoring system.

Importantly, because of the retrospective nature of the study, we could not account for variations in clinical practice over time and across neonatal centres. We also could not assess whether the environmental exposures of ambulance transfer added additional stress to these high-risk infants or if being born in a non-cooling centre modifies the treatment effect of TH.

## Conclusion

Implementation of active TH by transport and referring centres optimises the delivery of TH for infants treated for HIE who require transfer, most notably through earlier initiation and greater achievement of TT within 6 h of age. Investment in TH provision training and education to address disparities in TH care could potentially reduce brain injury and ease time pressures associated with HIE transfers for transport services. A larger national study validating our findings and associations with neurological outcomes could provide additional evidence for our study towards supporting national HIE recommendations.

## Supplementary information

Supplementary File

## Data Availability

Anonymised transport data was provided from the CenTre Neonatal Transport database. All data are included in this manuscript or the Supplementary Material.

## References

[1] Shipley, L., Gale, C. & Sharkey, D. Trends in the incidence and management of hypoxic-ischaemic encephalopathy in the therapeutic hypothermia era: a national population study. *Arch. Dis. Child. Fetal Neonatal Ed*. **106**, 529–534 (2021). 10.1136/archdischild-2020-320902 33685945

[2] Leslie, A. et al. Tracking national neonatal transport activity and metrics using the UK Neonatal Transport Group dataset 2012–2021: a narrative review. *Arch. Dis. Child. Fetal Neonatal Ed*. **109**, 460–466 (2024). 10.1136/archdischild-2023-325532 38272658

[3] Azzopardi, D. et al. Implementation and conduct of therapeutic hypothermia for perinatal asphyxial encephalopathy in the UK-analysis of national data. *PLoS ONE* **7**, e38504 (2012). 22719897 10.1371/journal.pone.0038504 PMC3374836

[4] Jacobs, S. E. et al. Cooling for newborns with hypoxic ischaemic encephalopathy. *Cochrane Database Syst. Rev*. CD003311 (2013). 10.1002/14651858.CD003311 14583966

[5] National Institute for Health and Care Excellence. Therapeutic hypothermia with intracorporeal temperature monitoring for hypoxic perinatal brain injury. https://www.nice.org.uk/guidance/ipg347/resources/therapeutic-hypothermia-with-intracorporeal-temperature-monitoring-for-hypoxic-perinatal-brain-injury-pdf-1899867578267077 (2010).

[6] Bourque, S. L. et al. A quality initiative for optimal therapeutic hypothermia during transport for neonates with neonatal encephalopathy. *Pediatr. Qual. Saf.* **3**, e056 (2018). 29732456 10.1097/pq9.0000000000000056 PMC5916477

[7] Hallberg, B., Olson, L., Bartocci, M., Edqvist, I. & Blennow, M. Passive induction of hypothermia during transport of asphyxiated infants: a risk of excessive cooling. *Acta Paediatr.* **98**, 942–946 (2009). 19484830 10.1111/j.1651-2227.2009.01303.x

[8] Eicher, D. J. et al. Moderate hypothermia in neonatal encephalopathy: efficacy outcomes. *Pediatr. Neurol.* **32**, 11–17 (2005). 15607598 10.1016/j.pediatrneurol.2004.06.014

[9] Sharma, A. Provision of therapeutic hypothermia in neonatal transport: a longitudinal study and review of literature. *Cureus* **7**, e270 (2015). 26180694 10.7759/cureus.270 PMC4494512

[10] Goel, N., Mohinuddin, S. M., Ratnavel, N., Kempley, S. & Sinha, A. Comparison of passive and servo-controlled active cooling for infants with hypoxic-ischemic encephalopathy during neonatal transfers. *Am. J. Perinatol.* **34**, 19–25 (2017). 27182995 10.1055/s-0036-1584151

[11] Chaudhary, R., Farrer, K., Broster, S., McRitchie, L. & Austin, T. Active versus passive cooling during neonatal transport. *Pediatrics* **132**, 841 (2013). 24144712 10.1542/peds.2013-1686

[12] Hagan, J. L. Meta-analysis comparing temperature on arrival at the referral hospital of newborns with hypoxic ischemic encephalopathy cooled with a servo-controlled device versus no device during transport.* J. Neonatal Perinat. M**ed*. **14**, 29–41 (2021). 10.3233/NPM-200464 32741783

[13] Lee, K. S. et al. Practice variations for therapeutic hypothermia in neonates with hypoxic-ischemic encephalopathy: an international survey. *J. Pediatr*. **274**, 114181 (2024). 10.1016/j.jpeds.2024.114181 38950817

[14] Lee, K.-S. Neonatal transport metrics and quality improvement in a regional transport service. *Transl. Pediatr.* **8**, 233–245 (2019). 31413957 10.21037/tp.2019.07.04 PMC6675684

[15] Shipley, L., Mistry, A. & Sharkey, D. Outcomes of neonatal hypoxic-ischaemic encephalopathy in centres with and without active therapeutic hypothermia: a nationwide propensity score-matched analysis. *Arch. Dis. Child. Fetal Neonatal Ed*. **107**, 6–12 (2021). 10.1136/archdischild-2020-320966 34045283

[16] Natarajan, G. et al. Effect of inborn vs. outborn delivery on neurodevelopmental outcomes in infants with hypoxic-ischemic encephalopathy: secondary analyses of the NICHD whole-body cooling trial. *Pediatr. Res.* **72**, 414–419 (2012). 22914450 10.1038/pr.2012.103 PMC3730811

[17] UK-NTG, Devon, C. & Jackson, A. UK Neonatal Transport Group Dataset. https://www.bapm.org/pages/ntg-dataset (2023).

[18] Redpath, S. et al. Effectiveness of therapeutic hypothermia on transport within a large geographical area. *Pediatrics* **141**, 723 (2018).

[19] Fenton, A. C. & Leslie, A. The state of neonatal transport services in the UK. *Arch. Dis. Child. Fetal Neonatal Ed.* **97**, F477–F481 (2012). 21948327 10.1136/archdischild-2011-300573

[20] British Association of Perinatal Medicine. Therapeutic hypothermia for neonatal encephalopathy. A BAPM framework for practice. https://www.bapm.org/resources/237-therapeutic-hypothermia-for-neonatal-encephalopathy (2020).

[21] Mistry, A., Shipley, L., Ojha, S. & Sharkey, D. Availability of active therapeutic hypothermia at birth for neonatal hypoxic ischaemic encephalopathy: a UK population study from 2011 to 2018. *Arch. Dis. Child. Fetal Neonatal Ed*. **507**, 597–602 (2022). 10.1136/archdischild-2021-322906 35428686

[22] Mistry, A., Leslie, A., Ojha, S. & Sharkey, D. Identifying neonatal transport research priorities: a modified Delphi consensus. *Arch. Dis. Child. Fetal Neonatal Ed*. **110**, 43–50 (2024). 10.1136/archdischild-2024-327213 38857987

[23] Azzopardi, D. V. et al. Moderate hypothermia to treat perinatal asphyxial encephalopathy. *N. Engl. J. Med.* **361**, 1349–1358 (2009). 19797281 10.1056/NEJMoa0900854

[24] British Association of Perinatal Medicine and Neonatal Transport Group. BAPM & NTG Neonatal Transport Dataset. https://www.bapm.org/pages/ntg-dataset (2016).

[25] Jayasinghe, D. Management of Neonatal Encephalopathy (North Hub) East Midland Operational Delivery Network. https://nuhp.koha-ptfs.co.uk/cgi-bin/koha/opac-retrieve-file.pl?id=134a14e79919711248823abe68dfcb18 (2021).

[26] Jackson, A. Neonatal Transport Group Dataset. https://www.bapm.org/pages/ntg-dataset (2019).

[27] Ratnavel, N. Evaluating and improving neonatal transport services. *Early Hum. Dev*. **89**, 851–853 (2013). 10.1016/j.earlhumdev.2013.09.004 24094330

[28] Gunn, A. J., Gunn, T. R., Gunning, M. I., Williams, C. E. & Gluckman, P. D. Neuroprotection with prolonged head cooling started before postischemic seizures in fetal sheep. *Pediatrics* **102**, 1098–1106 (1998). 9794940 10.1542/peds.102.5.1098

[29] Roelfsema, V. et al. Window of opportunity of cerebral hypothermia for postischemic white matter injury in the near-term fetal sheep. *J. Cereb. Blood Flow Metab.* **24**, 877–886 (2004). 15362718 10.1097/01.WCB.0000123904.17746.92

[30] Gunn, A. J. Cerebral hypothermia for prevention of brain injury following perinatal asphyxia. *Curr. Opin. Pediatr*. **12**, 111–115 (2000). 10.1097/00008480-200004000-00004 10763759

[31] Thoresen, M. et al. Time is brain: starting therapeutic hypothermia within three hours after birth improves motor outcome in asphyxiated newborns. *Neonatology* **104**, 228–233 (2013). 24030160 10.1159/000353948

[32] Youn, Y.-A. et al. The hospital outcomes compared between the early and late hypothermia-treated groups in neonates. * J. Matern. Fetal Neonatal Med.* **29**, 2288–2292 (2016). 26364841 10.3109/14767058.2015.1083548

[33] Wirrell, E. C., Armstrong, E. A., Osman, L. D. & Yager, J. Y. Prolonged seizures exacerbate perinatal hypoxic-ischemic brain damage. *Pediatr. Res.* **50**, 445–454 (2001). 11568286 10.1203/00006450-200110000-00005

[34] Shah, D. K. et al. Electrographic seizures are associated with brain injury in newborns undergoing therapeutic hypothermia. *Arch. Dis. Child Fetal Neonatal Ed.* **99**, F219–F224 (2014). 24443407 10.1136/archdischild-2013-305206

[35] Reynolds, P. South East Coast Neonatal Network Time=Brain Quality Improvment initiative. https://www.networks.nhs.uk/nhs-networks/south-east-coast-neonatal-network/time-brain/time-brain (2025).

[36] Mistry, A., Simpson, R. B., Ojha, S. & Sharkey D. Increasing availability of active therapeutic hypothermia for neonatal hypoxic ischaemic encephalopathy in the UK. *Arch. Dis. Child Fetal Neonatal Ed*. **110**, 430–431 (2025). 10.1136/archdischild-2024-328119 39663144

[37] British Association of Perinatal Medicine. Service and quality standards for provision of neonatal care in the UK. https://www.bapm.org/resources/service-and-quality-standards-for-provision-of-neonatal-care-in-the-uk (2022).

[38] Stafford, T. D., Hagan, J. L., Sitler, C. G., Fernandes, C. J. & Kaiser, J. R. Therapeutic hypothermia during neonatal transport: active cooling helps reach the target. *Ther. Hypothermia Temp. Manag.* **7**, 88–94 (2016). 27676120 10.1089/ther.2016.0022

[39] Lumba, R., Mally, P., Espiritu, M. & Wachtel, E. V. Therapeutic hypothermia during neonatal transport at regional perinatal centers: active vs. passive cooling. *J. Perinat. Med.* **47**, 365–369 (2019). 30530909 10.1515/jpm-2018-0302

[40] Austin, T. et al. Neonatal brain magnetic resonance imaging: clinical indication, acquisition and reporting. *Arch. Dis. Child. Fetal Neonatal Ed*. **109**, 348–361 (2024). 10.1136/archdischild-2023-326747 38373753

[41] Parmentier, C. E. J., de Vries, L. S. & Groenendaal, F. Magnetic resonance imaging in (near-)term infants with hypoxic-ischemic encephalopathy. *Diagnostics* **12**, 645 (2022). 10.3390/diagnostics12030645 PMC8947468 35328199

[42] Bednarek, N. et al. Impact of therapeutic hypothermia on MRI diffusion changes in neonatal encephalopathy. *Neurology* **78**, 1420–1427 (2012). 10.1212/WNL.0b013e318253d589 PMC3345786 22517107

