# Supplementary material for: Implementation of active therapeutic hypothermia across a regional transport network for infants transferred for neonatal encephalopathy
Source: Pediatr Res. 2025 Jun 27;99(2):718–27. doi: 10.1038/s41390-025-04248-x (PMC12956594; doi:10.1038/s41390-025-04248-x)
Supplement: Supplementary file 1 — Supplementary File [file 41390_2025_4248_MOESM1_ESM.pdf]

Supplemental file

Title

Implementation of active therapeutic hypothermia across a regional transport network for infants transferred for neonatal encephalopathy

Authors: Dr Aarti Mistry[1], Dr Nora Imolya[2], Dr Jack Fletcher[3], Dr Dharmapuri Sobithadevi[3] Dr Davina Sham[4], Dr Ben James Baucells[4], Dr Julia Edwards[2], Dr Arthi Lakshmanan[5], Dr Andrew Currie[4], Dr Andrew Leslie[1], Professor Shalini Ojha[1], Professor Don Sharkey[1]

Corresponding Author: Professor Don Sharkey, Centre for Perinatal Research (CePR), School of Medicine, E floor, East Block, University Hospital, Derby Rd, Nottingham, NG7 2UH, UK. Don.Sharkey@nottingham.ac.uk. Tel no. +44 1158230611.

Affiliation 1: Centre for Perinatal Research (CePR), School of Medicine, University of Nottingham

Affiliation 2: Nottingham Centre for Neonatal Care, Nottingham University Hospitals NHS Trust, Nottingham, UK

Affiliation 3: Neonatal service, University Hospitals Derby and Burton NHS Trust, Derbyshire, UK

Affiliation 4: CenTre Neonatal Transport Service, University Hospitals of Leicester NHS Trust, UK

Affiliation 5: Neonatal Service, University Hospitals of Coventry and Warwickshire NHS Trust

Supplemental file

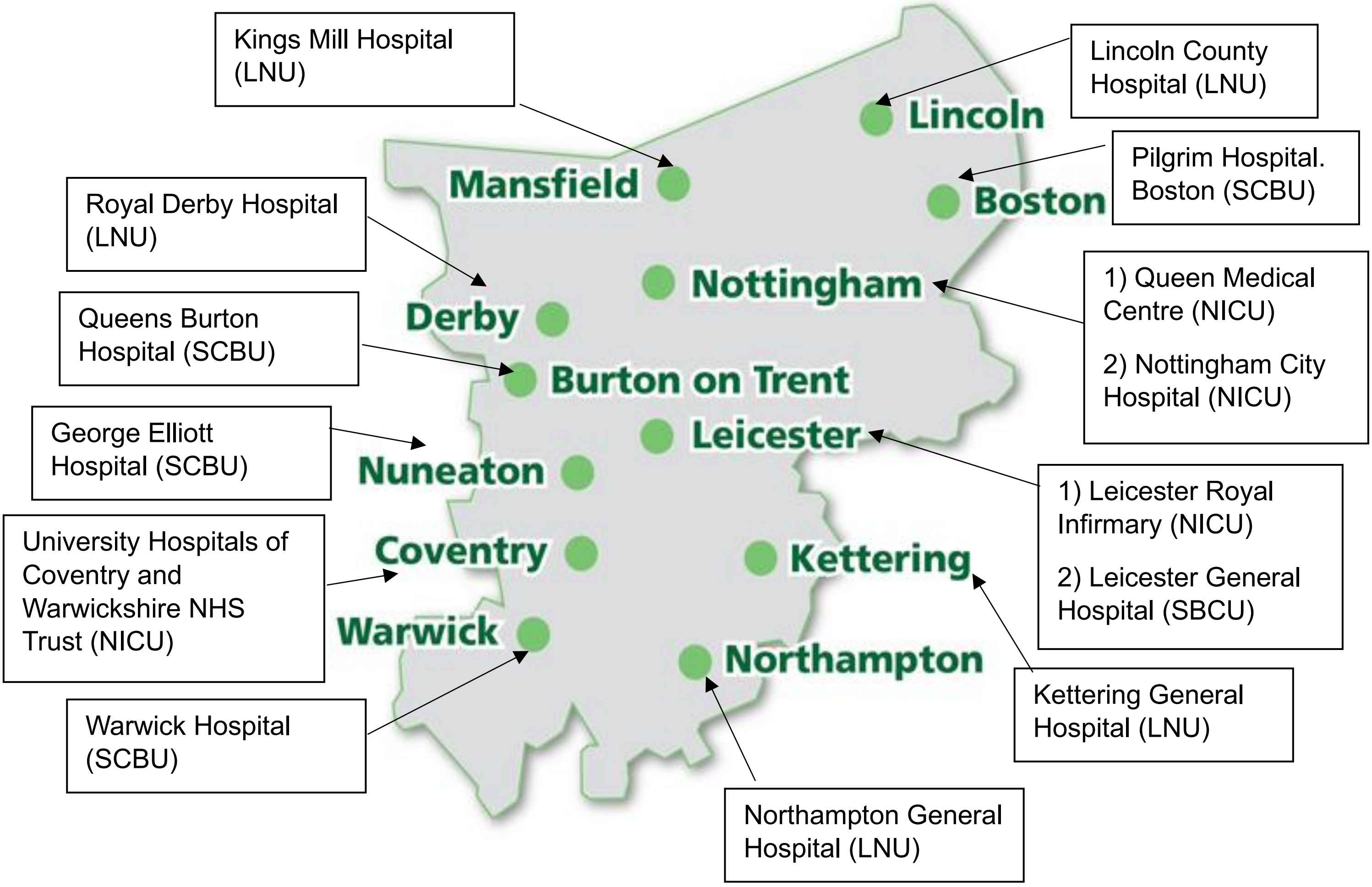

(Figure 1)

Depicts the areas covered by the Trent perinatal Central neonatal network.[(1)]

Units within this network are listed below by level of neonatal unit:

Neonatal Intensive Care Units (NICU- Level 3) and Tertiary cooling centres: Queens Medical Centre, Nottingham City Hospital, University Hospitals of Coventry and Warwickshire NHS Trust, Leicester Royal Infirmary

Local Neonatal Units (LNU-Level 2): Lincoln County Hospital, Royal Derby Hospital, Kings Mill Hospital, Kettering General Hospital, Northampton General Hospital

Special Care Baby Units (SCBU-Level 1): Leicester General Hospital, Queens Burton Hospital, Pilgrim Boston Hospital, George Elliot Hospital, Warwick Hospital

(Table 1) Annual number of transfers undertaken by CenTre transport and HIE transfers from 2011-2021

Year	Total number transfers by CenTre per year	Total number of completed HIE transfers (% of annual transfer)	Number of uplift transfers	% of HIE transfers to uplift transfers.
2011/12	1298	42 (3.2%)	508	8.3%
2012/13	1407	46 (3.3%)	549	8.4%
2013/14	1464	41 (2.8%)	545	7.5%
2014/15	1582	40 (2.5%)	608	6.6%
2015/16	1660	43 (2.6%)	635	6.8%
2016/17	1627	45 (2.8%)	654	6.9%
2017/18	1612	31 (1.9%)	600	5.2%
2018/19	1646	36 (2.2%)	686	5.2%
2019/20	1554	38 (2.4%)	640	5.9%
2020/21	1403	32 (2.3%)	587	5.5%
Total	15253	394 (2.6%)	6012	6.6%

(Table 2) CenTre ambulance and cooling equipment procurement timeline

Timeframe (Year)	Specification of Ambulance service procurement	Cooling equipment procurement
2010-mid 2012	No dedicated crew or vehicles	Passive cooling during transfer
2012-end 2013	Dedicated crews not vehicles	
End 2013-2014	Dedicated crews and 2 Vehicles for North and South -Transition	
2015 to Present	Dedicated crews and vehicles secured	June 2015 access to servocontrolled cooling devices for ambulances (CritiCool®)

(Table 3) All variables collected and created from the database, type of variable and how they were defined.

Variable	Type of variable	Definition
Gestation	Continuous	Completed gestation weeks
Sex	Categorical	Male or Female
Birth order	Categorical	Singleton or Twin
Birthweight	Continuous	Weight in grams
Actual centile	Continuous	Birth weight centile
Referring centre	Categorical	Centre referring infant for transfer
Tertiary cooling centre	-	NICU providing active therapeutic hypothermia for cooling
Level of unit		
SCBU	Categorical	Special care baby unit
LNU		Local neonatal unit
NICU		Neonatal intensive care
Antenatal events		
IUGR	Binary	Intrauterine growth restriction - is a fetal weight that is below the 10th percentile for gestational age as determined through an ultrasound. Intrauterine Growth Restriction is also known as Small-for-Gestational-Age (SGA) or fetal growth restriction
LGA	Binary	Large for gestation age, birthweight >90th centile for gestation age
Reduce fetal movements	Binary	Reduced fetal movements during pregnancy
Preeclampsia /PET	Binary	Preeclampsia toxaemia.<br>A condition that occurs in the second half of pregnancy, associated with high blood pressure and protein in the urine. Mother maybe on anti-hypertensive agents during pregnancy such (nifedipine, labetalol, magnesium sulphate)
Gestational /IDDM	Binary	Gestational or IDDM If a woman at any point in pregnancy has either:<br>• a fasting plasma glucose level of 5.6 mmol/litre or above or<br>• a 2-hour plasma glucose level of 7.8 mmol/litre or above. Mother may have raised HbA1C of 42 or be commenced on metformin or insulin during pregnancy
APH, Placenta issue	Binary	**Antepartum Haemorrhage:** bleeding from or into the genital tract, occurring from 24+0 weeks of pregnancy and prior to the birth of the baby<br>**Placenta Praevia:** A condition where the placenta covers all or part of the cervix. Diagnosed through antenatal scans<br>**Placental abruption**: The premature separation of a normally located placenta from the uterine wall that occurs before delivery of the fetus. Abruption may be revealed, when blood escapes through the vagina, or concealed, when the bleeding occurs behind the placenta, with no evidence of bleeding from the vagina
Maternal infection	Binary	This based presence of one of the following:<br>**Maternal Pyrexia** in labour > 38.5°C<br>**Maternal sepsis:** IV antibiotics given to mother for confirmed or suspected invasive bacterial infection (such as septicaemia) at any time during labour, or in 24-hr periods before and after the birth (this does not refer to IAP)<br>**Chorioamnionitis**: An infection inside the uterus affecting the membranes (called the chorion and amnion) which surround the amniotic fluid. Identified clinically or Positive bacterial /Viral swab of placenta tissue<br>**UTI**: A lower urinary tract infection (UTI) is an infection of the bladder (also known as cystitis) usually caused by

		bacteria from the gastrointestinal tract.<br>Upper UTI is infection of the upper part of the urinary tract - the ureters and kidneys (pyelonephritis).<br>Confirmed bacteraemia on maternal urine sample or positive urine dipstick with (leucocytes and nitrites)
PROM	Binary	Prolonged rupture of membranes of > 18hrs
Mode of delivery		
NVD<br>Instrumental<br>EM-LSCS in labour<br>EM-LSCS not in labour<br>EL-LSCS not in labour	Categorical	Normal vaginal delivery<br>Ventouse, forceps, kiwi delivery<br>Emergency low caesarean section in labour<br>Emergency lower caesarean section not in labour<br>Elective lower caesarean section not in labour
Onset of labour	Binary	Onset of labour- spontaneous, induced or not in labour
Presentation at birth	Binary	Presentation of head during labour e.g Breech, cephalic, transverse, face
Intrapartum event		
Shoulder dystocia	Binary	Shoulder dystocia is when the baby's head has been born but one of the shoulders becomes stuck behind the mother's pubic bone, delaying the birth of the baby's body
Cord prolapse	Binary	Descent of the umbilical cord through the cervix alongside (occult) or past the presenting part (overt) in the presence of ruptured membranes. Cord presentation is the presence of the umbilical cord between the fetal presenting part and the cervix, with or without membrane rupture
Malposition	Binary	Malposition: abnormal positions of the vertex of the fetal head (with the occiput as the reference point) relative to the maternal pelvis<br>Malpresentation: fetal malpresentation refers to a fetus with a fetal part other than the head engaging the maternal pelvis. Example Face, Brow, Chin
Failure to progress	Binary	Prolonged or delayed second stage of labour
Fetal distress in labour	Binary	This could be based on an abnormal fetal blood sample gas, or evidence of pathological CTG (reduced variability, increasing number of decelerations, fetal tachycardia)
Fetal Bradycardia	Binary	Evidence of fetal bradycardia on CTG
Significant meconium	Binary	Dark green or black amniotic fluid that is thick or tenacious or any meconium-stained amniotic fluid containing lumps of meconium or visualised on direct suction oropharynx
Resuscitation		
No support	Binary	No resuscitation required at birth
Face mask only	Binary	Face mask ventilation as part of resuscitation at birth
Intubation	Binary	Intubation as part of resuscitation at birth
Chest compressions	Binary	Chest compressions as part of resuscitation at birth
Drugs-adrenaline	Binary	Use of adrenaline as part of resuscitation at birth
APGARS	Continuous	APGARS @1 min, @5 mins and @10mins
Duration of resuscitation	Continuous	Time from birth to completion of resuscitation period
Cord Venous Ph	Continuous	Cord Ph Venous gas
Evidence of encephalopathy	Binary	Evidence of encephalopathy- presence of all three (reduced activity/conscious level, absent reflexes, abnormal, tone) or seizures after birth
NICU Management		
No respiratory support	Binary	Infants requiring no respiratory support or just nasal cannula during NICU tertiary cooling centre stay
Non-Invasive Ventilation	Binary	Infants requiring non-invasive respiratory support i.e. CPAP and High Flow during NICU tertiary cooling centre stay
Mechanical ventilation	Binary	Infants requiring invasive mechanical ventilation including HFOV during NICU tertiary cooling centre stay
Days of ventilation	Binary	Days of mechanical ventilation received
NICU Inotropes	Binary	Use of inotropes during NICU management
NICU Anticonvulsants	Binary	Use of anticonvulsants during NICU management

NICU Seizures	Binary	Presence of seizures both clinical or electrically and using anticonvulsants as proxy for seizures on NICU
CFAM Abnormal	Binary	Abnormal Cerebral function ambulatory monitoring. Infant identified to have a moderate or severe abnormality on tracing. This could mean seizures, burst suppression or discontinuous tracing
Evidence of Brain injury on MRI	Binary	Evidence of any hypoxic ischaemic brain injury on Magnetic Resonance Imaging (MRI). Those died without MRI were presumed to have an abnormal MRI. MRI imaging consisted of T1, T2 and diffusion weighted images
Day MRI performed	Continuous	Day of infant's life to which MRI was performed
Completed cooling 72hrs	Binary	Completing active therapeutic servocontrolled hypothermia for 72hrs
Intended to completed cooling	Binary	Presumes that the infant would have completed active therapeutic servocontrolled hypothermia for 72hrs, however died prior to completion, or too sick to continue cooling
Grade of Hypoxic ischaemic encephalopathy	Categorical	Mild, Moderate, Severe as documented on Badger or assessed through medical documentation using the SARNAT scoring
Final discharge destination -Died	Categorical	Death at discharge
Final discharge destination - Home	Categorical	Home at discharge
Final discharge destination - Hospice	Categorical	Hospice at discharge
Final discharge destination -Other	Categorical	Transfer to other specialist care i.e. PICU
Oral Feeds at discharge	Binary	Oral feeds defined as bottle or breastfeeds. No oral feeds would refer to nasogastric feeds, PEG or JEG feeds.
Survival at discharge	Binary	Survival at the point of discharge
Survival without brain injury	Binary	Survival with evidence of brain injury on MRI. Composite outcome. Those that died are presumed to have evidence of brain injury on MRI
Survival without seizures	Binary	Survival without seizures. Composite outcome. Seizures are defined as either clinical or electrical or using anticonvulsants as a proxy
Transport specific		
Age of referral (min)	Continuous	Age of infant when referred to transport service by referring centre. Calculated from time of birth and transport time, or ascertained from transport notes
Age to departing base (min)	Continuous	Age of infant to when transport team depart from base
Age to arrival ref centre (min)	Continuous	Age of infant to when transport team arrive at referring centre
Age to departure ref centre (min)	Continuous	Age of infant when transport team depart from base
Age to admission receiving centre (min)	Continuous	Age of infant when they are admitted/arrive at receiving tertiary cooling centre
Mobilisation time (min)	Continuous	Time from referral to departing transport base also referred to as despatch time
Response time (min)	Continuous	Time from referral to arrival at referring centre
Stabilisation time (min)	Continuous	Time from arriving and departing referring centre
Mobilisation time < 60min	Binary	Achieving time from referral to departing base within 60mins. (yes or no)
Response time <3.5hr	Binary	Achieving time from referral to arrival at referring centre within 3.5hrs (yes or no)
Age to TT	Continuous	Age to reaching therapeutic target temperature range (33-34°C)
TT <6hrs	Binary	Therapeutic target temperature in range less than 6hrs of age
TT 0-3hrs	Binary	Therapeutic target temperature in range between 0-3hrs of age
TT > 6hrs	Binary	Therapeutic temperature in range > 6hrs of age
Hypocarbia in ventilated infants	Continuous	PCO2<4kpa on end of transfer gas

Temperature at transfer timepoint	Continuous	Temperatures measured on arrival to referring centre, departure from referring centre and admission to tertiary cooling centre. Temperatures were recorded using rectal monitoring at departure and admission. Reading at arrival to referral centre may have been rectal or axillary
Transfer management:		
No respiratory support	Binary	No respiratory support required during transport
Non-Invasive Ventilation	Binary	Non-invasive respiratory support i.e. CPAP or High Flow support during transport
Mechanical Ventilation	Binary	Mechanical ventilation including HFOV during transport
Inotropes	Binary	Inotropes during transport
Nitric oxide	Binary	Use of nitric oxide during transport
Seizures/anticonvulsants	Binary	Seizures (Both clinical or electrical) or use of anticonvulsants prior to completing transfer

(Table 4)

Presents the transfer distances across comparison cohorts and when target temperature (33-34°C) reached within 6 hours of age. Comparisons between cohort 1 and 2, and cohort 2 and 3, are presented. Missing n (%) based on denominator n=315. Mann Whitney test used to compare statistical differences between groups. Statistical significance defined P<0.05. Data are median (IQR) and *Min / Max

	1.Base^{Pass}/Transport^{Pass} n=155	2.Base^{Pass}/Transport^{Act} n=128	3.Base^{Act}/Transport^{Act} n=32	1 vs 2 P Value	2 vs 3 P Value	Missing n (%)
**Transfer distance (km)**	52.5 (20.4, 86.6)	41.5 (20.4, 69.0)	34.4 (26.4, 34.4)	0.26	0.10	-
	*4.9 / 129.4	4.9 / 119.5	8.4 / 69.0	-	-	-
**Target temperature**						
**<6 hours of age**	49.6 (20.4, 69.2)	46.6 (20.4, 69.0)	34.4 (26.4, 34.4)	0.66	0.13	8 (2.5)

Reference

1. CenTre Neonatal Transport -Our Hospitals 2024 [Available from: https://www.centreneonataltransport.nhs.uk/about-us/our-hospitals/. Accessed January 2025]
